# Pan-cancer and experimental analyses reveal the immunotherapeutic significance of CST2 and its association with stomach adenocarcinoma proliferation and metastasis

**DOI:** 10.3389/fimmu.2024.1466806

**Published:** 2025-01-24

**Authors:** Dan Huang, Jing Li, Zhijun He, Wenjing Liang, Likun Zhong, Jun Huang, Yinteng Wu, Shijian Zhao

**Affiliations:** ^1^ Department of Gastroenterology, The People’s Hospital of Guangxi Zhuang Autonomous Region (Guangxi Academy of Medical Sciences), Nanning, Guangxi, China; ^2^ Department of Orthopedic and Trauma Surgery, The First Affiliated Hospital of Guangxi Medical University, Nanning, Guangxi, China; ^3^ Department of Cardiology, The Affiliated Cardiovascular Hospital of Kunming Medical University (Fuwai Yunnan Cardiovascular Hospital), Kunming, Yunnan, China

**Keywords:** cystatin 2 (CST2), pan-cancer, stomach adenocarcinoma (STAD), the tumor microenvironment (TME), immunotherapy, single-cell

## Abstract

**Purpose:**

Cystatin 2 (CST2) is a cysteine protease inhibitor, and recent research suggests its potential involvement in cancer development. However, its role in the occurrence, progression, and prognosis of pan-cancer has not been systematically investigated.

**Materials and methods:**

This study comprehensively analyzes the differential expression of CST2 in pan-cancer. The expression distribution patterns of CST2 were examined using single-cell datasets. Furthermore, we conducted a comprehensive evaluation of the correlation between CST2 expression and various factors. These factors include prognosis, immune cell infiltration, immune-related genes, mutations, methylation, tumor mutation burden (TMB), and microsatellite instability (MSI). In addition, we analyzed the sensitivity of drugs dependent on CST2 expression. We utilized gene set enrichment analysis (GSEA) analysis to explore the biological functions of CST2 across different cancer types. Finally, in gastric cancer cell lines, we will investigate the impact of CST2 knockout on expression levels, clonal proliferation, cell apoptosis, and cell migration.

**Results:**

CST2 exhibits abnormal overexpression in multiple tumors. Single-cell analysis reveals high expression of CST2 in fibroblasts. CST2 is closely associated with prognosis, immune cell infiltration, immune-related genes, mutations, methylation, TMB, and MSI. Enrichment analysis demonstrated a significant correlation between CST2 and immune-related pathways. In stomach adenocarcinoma (STAD), CST2-related risk models are associated with prognosis and demonstrate strong predictive capabilities, while also being closely linked to the immune microenvironment. Drug sensitivity analysis indicates the correlation between CST2 and 21 chemotherapy drugs. Finally, experimental validation revealed significantly elevated expression of CST2 in STAD, indicating its role as a driver gene in regulating malignant cell proliferation and migration.

**Conclusion:**

CST2 serves as a potential tumor immune biomarker, playing a critical facilitating role in the proliferation and migration processes of STAD.

## Introduction

Cancer remains a leading cause of global human mortality (1). Given its high incidence and mortality rates, the pursuit of more valuable and targeted biomarkers for early diagnosis, treatment, and prevention is of paramount importance and urgency. The involvement of the tumor immune microenvironment in cancer initiation and progression has been gradually elucidated. Currently, molecular targeted therapies are progressively being applied in clinical practice. The discovery of biomarkers has significantly accelerated the development of anti-cancer drugs.

With the widespread adoption of high-throughput sequencing technologies and the improvement of tumor data sharing platforms, pan-cancer research is receiving increasing attention. By combining and analyzing cancers originating from different organs, pan-cancer studies can provide a deeper and broader understanding of common oncogenic signaling pathway characteristics, allowing researchers to focus on datasets with relatively larger sample sizes. Larger sample sizes enhance the statistical power of the data and make it easier to identify cancer-associated genomic alterations, potentially leading to the discovery of previously unidentified drug targets. Additionally, new tumor classification methods based on an understanding of common signaling pathway features can help certain cancer patients receive more personalized treatments, increasing the likelihood of disease relief (2).

Cystatin 2 (CST2) is a gene encoding a protein that belongs to the cysteine protease inhibitor superfamily (3). Previous studies have indicated that CST2 can predict disease progression in certain non-tumor conditions (4, 5). A recent study discovered that CST2 is overexpressed in pancreatic cancer, functioning as an oncogene. Knockdown of CST2 in pancreatic cancer inhibits tumor cell proliferation, migration, and invasion, while also suppressing the activation of the PI3K/AKT signaling pathway (6). Elevated levels of CST2 in colorectal cancer are associated with shortened overall survival in patients (7). The upregulation of CST2 has been linked to breast cancer development (8). In gastric cancer samples, CST2 is upregulated, enhancing tumor cell growth, migration, and invasion by regulating epithelial-mesenchymal transition (EMT) and the TGF-β1 signaling pathway, leading to poor prognosis in patients (9). CST2 may participate in prostate cancer metastasis by modulating the EMT signaling pathway (10). CST2 is associated with overall survival rate (OS) in hepatocellular carcinoma (11). High expression of CST2 promotes bone metastasis occurrence (12), which is common in solid tumors. Therefore, dysregulation of CST2 is implicated in human cancer. Nevertheless, there remains a need for a comprehensive understanding of CST2’s role in pan-cancer. Hence, further exploration of the mechanisms underlying CST2’s involvement in tumors holds significant importance in providing new directions and strategies for clinical cancer treatment.

By integrating multiple databases, we conducted an analysis of CST2 expression levels in pan-cancer and its relationship with tumor-infiltrating immune cells, immune-related genes, mutations, DNA methylation, tumor mutation burden (TMB), microsatellite instability (MSI), and their impact on patient prognosis. The results demonstrate that CST2 overexpression in pan-cancer contributes to carcinogenesis and is closely associated with the tumor immune microenvironment (TIM). Furthermore, we performed molecular biology validation in gastric cancer to further confirm the oncogenic role of CST2. In summary, CST2 represents a promising therapeutic target in cancer treatment and serves as a potential biomarker for predicting immunotherapy efficacy and prognosis.

## Materials and methods

### CST2 expression analysis in human pan-cancer

CST2 expression data in 35 normal tissues were obtained and downloaded from the Genotype-Tissue Expression (GTEx, https://commonfund.nih.gov/GTEx) database. Additionally, CST2 expression data from 31 tumor cell lines were obtained from the Cancer Cell Line Encyclopedia (CCLE, https://portals.broadinstitute.org/ccle/) database. By combining the data from normal tissues in the GTEx database and the cancer genomic atlas from The Cancer Genome Atlas (TCGA, https://www.cancer.gov/about-nci/organization/ccg/research/structural-genomics/tcga), the differential expression of CST2 between cancer tissues and normal tissues was analyzed. Data from 15 different types of cancer were retrieved from the TCGA database. We also investigated the expression levels of CST2 in different clinical stages.

### Single-cell analysis

To further analyze the single-cell expression distribution pattern of CST2 in pan-cancer, we analyzed various single-cell datasets, including BRCA, BTCC, CAC, CCRCC, CRPC, ESCC, NNSC, ICC, NPC, NSCLC, OV, PDAC, STAD, UCEC. These data single-cell data were obtained from the Single-cell and Spatially-resolved Cancer Resources (SCAR) database (http://scaratlas.com).

### Prognostic assessment in pan-cancer

Utilize the R package “survival” to establish a univariate Cox regression model. Evaluate the prognostic value of CST2 in different tumor types based on Overall Survival (OS), Progression Free Interval (PFI), Disease Specific Survival (DSS), and Disease Free Interval (DFI). Plot Kaplan-Meier survival analysis curves to depict the relationship between CST2 expression and OS, DSS, disease-free interval (DFS), and progression-free interval (PFS) specifically in STAD. The Gene Expression Omnibus (GEO, https://www.ncbi.nlm.nih.gov/geo/) database was used to validate the prognostic analysis of CST2 in STAD. We also analyzed the prognostic significance of CST2 in the immunotherapy cohort. Prognostic evaluation criteria include Hazard Ratio (HR), 95% confidence intervals, and p-values considered statistically significant when p<0.05.

### Immunological analyses

The TIMER2.0 database (https://cistrome.shinyapps.io/timer/) and Biomarker Exploration for Solid Tumors database (https://rookieutopia.hiplot.com.cn/app_direct/BEST/) can be employed to analyze tumor immune cell infiltration., which can be utilized for correlation analysis between CST2 and various types of immune cells. Visualization of the analysis results can be done using the “ggplot2” R package. Furthermore, the ESTIMATE algorithm can be applied to calculate the ESTIMATEScore, ImmuneScore, and StromalScore for different tumor types. Spearman algorithm can be used to determine the correlation coefficients between CST2 expression and these three scoring systems.

### Immunological correlation analysis

In each sample, the expression data for 130 immune-related genes were extracted, including 38 chemokines, 43 immunostimulators, 18 receptors, 8 immune checkpoint genes, and 23 immunoinhibitors. Subsequently, the Spearman algorithm was utilized to calculate the correlations between CST2 and individual immune-related genes.

### The evaluation of CST2 in pan-cancer regarding mutations, methylation, TMB, and MSI

The CBioPortal database (https://www.cbioportal.org/) was used to explore the mutation characteristics and locations of CST2 in tumors. To analyze the relationship between CST2 expression levels and the methylation status of its promoter region, we utilized the TCGA database and visualized the results using the “ggplot2” R package. We employed Spearman’s test in the TCGA database to assess the correlation between CST2 expression and TMB as well as MSI across different tumor types. The correlation results were then visualized using the “fmsb” R package.

### Enrichment analysis of CST2

To gain further insights into the biological functions and molecular mechanisms of CST2, we conducted Gene Set Enrichment Analysis (GSEA) using the R package ClusterProfiler. In this analysis, we employed the hallmark gene set from the Molecular Signatures Database (MSigDB), which consists of 50 gene sets associated with key cancer pathways. For each pathway, we calculated the Normalized Enrichment Score (NES) and False Discovery Rate (FDR) to assess the enrichment of CST2. Statistical significance was determined at a p-value < 0.05.

### Drug sensitivity analysis

To investigate the correlation between CST2 expression levels and drugs, we employed two databases. Firstly, the CellMiner database (https://ngdc.cncb.ac.cn/databasecommons/database/id/6092) allowed us to analyze gene expression profiles and their association with drug response. Furthermore, we explored the expression levels of CST2 in gastric cancer and its correlation with drugs using the Genomics of Drug Sensitivity in Cancer (GDSC, https://www.cancerrxgene.org) database and Cancer Therapeutics Response Portal (CTRP, https://portals.broadinstitute.org/ctrp.v2.1/) database.

### Cell culture and transfection

Human STAD cell lines MKN-45 and SGC-7901 were obtained from X-Y Biotechnology and maintained in DMEM medium supplemented with 10% fetal bovine serum at 37ru in a cell incubator with 5% CO2. For transfection experiments, 3 × 10^5 thyroid cancer cells were seeded into 6-well dishes and cultured for 24 hours to allow for cell attachment. Transfections were performed using Lipofectamine 3000 reagent (Thermo Fisher Scientific) according to the manufacturer’s protocol. Biological experiments were conducted following the appropriate transfection period. The siRNA sequences used in this study are as follows: si-CST2-1: 5’-GCUCCUCGAGACAUGUAAU-3’ (targeting 70-90bp downstream of the start codon, the Antisense strand: 5’-AUUACAUGACUCGAGAAGC-3’), si-CST2-2: 5’-GGACGAGGUUCUUGUAAAU-3’ (targeting 150-170bp downstream of the start codon, the Antisense strand: 5’-AUUUCCAAGAACCACGUCC-3’).

### Plate clone formation assay

Cells were collected and resuspended to obtain a single-cell suspension. The cells were seeded in 35 mm cell culture dishes at a density of 200 cells per dish. The medium was replaced every 2–3 days, with half of the medium being replaced each time. After approximately 14 days, the colonies were analyzed. Clones with more than 20 cells were counted under a microscope. Subsequently, the colonies were fixed with methanol and stained using a 1% crystal violet staining solution (G1063, Solarbio, China).

### Cell migration assay

During the transwell invasion experiment, first add 150 µL of DMEM culture medium with an additional 10% fetal bovine serum (FBS) to the lower chamber. Then, seed a total of 2 × 10^4 MKN-45 and SGC-7901 cells in the upper chamber of the transwell device. After 24 hours of incubation, carefully remove the non-invasive cells on the membrane surface. Next, fix the cells that have successfully penetrated the membrane and invaded the lower surface, followed by staining with crystal violet dye (G1063, Solarbio, China). Finally, use a microscope to capture images of the stained invasive cells and quantify the number of invasive cells by counting the cells in three randomly selected fields per well.

### Flow-cytometric analysis

To evaluate cell apoptosis, first seed MKN-45 and SGC-7901 cells into a 6-well plate. Then, transfect the cells with si-NC and si-CST2 separately for 24 hours. After transfection, digest the cells with trypsin and wash them twice with pre-chilled PBS (4S-c to remove residual culture medium and enzymes. The washed cells are resuspended in binding buffer, and according to the instructions provided by Absin company (China) using the Annexin V-FITC/PI Cell Apoptosis Detection Kit (abs50001-25T), stain the cells with Annexin V-FITC and propidium iodide (PI). The stained cells are transferred to tubes specifically designed for flow cytometry analysis and cell apoptosis is detected using a BD FACSCalibur flow cytometer. Data collection and analysis are performed using FlowJo software. The percentage of apoptotic cells is accurately calculated by distinguishing Annexin V-positive and PI-negative cells (early apoptosis) from Annexin V-positive and PI-positive cells (late apoptosis).

### Western blot

The cells are first washed with ice-cold PBS, followed by lysis using a protein extraction kit (20127ES60, Yeasen). The collected cell samples are centrifuged at 700g for 10 minutes at 4°C. After centrifugation, the supernatant is transferred to a new Eppendorf tube to avoid particle contamination. Subsequently, at 4tbs the supernatant is centrifuged again at 14,000g for 30 minutes to pellet cell membrane debris. The cell pellet is then resuspended in 200 µL of BCA protein assay kit B solution (containing PMSF), vortexed for 5 seconds, and placed on ice for 5 minutes. This step is repeated twice. After the initial step, centrifugation is performed at 14,000g for 5 minutes at 4tn to collect the supernatant containing the membrane proteins. The protein concentration is determined using the BCA protein assay kit (20201ES76, Yeasen) following standard operating procedures. Subsequently, total protein is separated by electrophoresis on a 4-20% Bis-Tris gel (Genscript China) and transferred to a PVDF membrane (ISEQ00010, Millipore). The membrane is initially incubated at room temperature in TBST buffer (Tris-buffered saline with Tween) containing 5% skim milk for 1 hour to block non-specific binding. Following the blocking step, it is further incubated overnight at 4te with primary antibodies (including Anti-CST2, 1:1000, abs115674, Absin; and Anti-GAPDH, 1:2000, ab8245, Abcam) diluted in TBST with 0.5% skim milk. After the primary antibody incubation, the membrane is washed three times with TBST and subsequently incubated with HRP-conjugated secondary antibodies (34201ES60, 34101ES60, Yeasen) diluted in TBST at room temperature for 1 hour. Immunoreactive bands are detected using an ECL Western blotting substrate (36208ES60, Yeasen). Finally, the density of the bands is quantitatively analyzed using ImageJ software.

## Results

### Aberrant expression of CST2 in human pan-cancer

**Figure 1:**
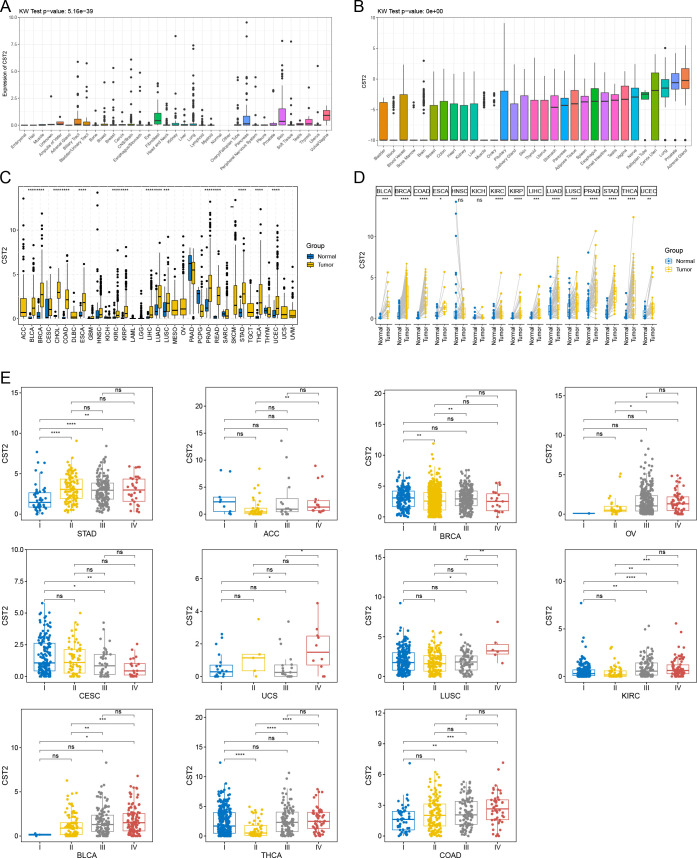
Cystatin 2 (CST2) aberrant expression and its correlation with clinical stages in human pan-cancer **(A)** Expression of CST2 across 35 tissues in the Genotype-Tissue Expression (GTEx) database. **(B)** Expression of CST2 in 31 tumor cell lines in the Cancer Cell Line Encyclopedia (CCLE) database. **(C)** Differential expression of CST2 between cancerous and normal tissues in The Cancer Genome Atlas (TCGA) database. **(D)** Aberrant overexpression of CST2 in 13 types of cancer based on analysis of the GTEx and TCGA databases. **(E)** Variations in CST2 expression levels among distinct clinical stages in STAD, ACC, BRCA, OV, CESC, UCS, LUSC, KIRC, BLCA, THCA, and COAD. (*p < 0.05, **p < 0.01, ***p < 0.001, and ****p < 0.0001).

Initially, we analyzed the expression of CST2 across 35 normal tissues utilizing the GTEx database. As depicted in **Figure 1A**, we observed relatively high expression levels of CST2 in Fibroblast, Skin, and Vagina tissues. Additionally, we accessed data from the CCLE database to investigate CST2 expression in 31 tumor cell lines. As shown in **Figure 1B**, CST2 was expressed in all 26 types of tumor cells examined. To determine the differential expression of CST2 between tumor and normal tissues, we conducted an analysis using the TCGA database for 33 different cancer types. The results revealed significantly higher expression levels of CST2 in BLCA, BRCA, CHOL, COAD, ESCA, KIRC, KIRP, LIHC, LUAD, LUSC, PRAD, READ, STAD, THCA, and UCEC tissues when compared to normal tissues (**Figure 1C**). Considering the limited number of normal samples available in TCGA, we integrated data from the GTEx and TCGA databases to analyze CST2 expression differences in 15 tumor types. We found that CST2 was significantly upregulated in 13 tumor types, including BLCA, BRCA, COAD, ESCA, KIRC, KIRP, LIHC, LUAD, LUSC, PRAD, STAD, THCA, and UCEC, as compared to their respective normal tissues (**Figure 1D**). These findings indicate the aberrant overexpression of CST2 in human pan-cancer.

### Different clinical stages

Through comprehensive analysis and evaluation, we have discovered distinct expression patterns of CST2 across different clinical stages in several tumor types. Specifically, higher levels of CST2 expression were observed in the advanced stages of STAD, ACC, BRCA, OV, UCS, LUSC, KIRC, BLCA, THCA, and COAD (**Figure 1E**). However, in the case of CESC, CST2 exhibited lower expression levels in the advanced stages.

### Single-cell analysis

**Figure 2:**
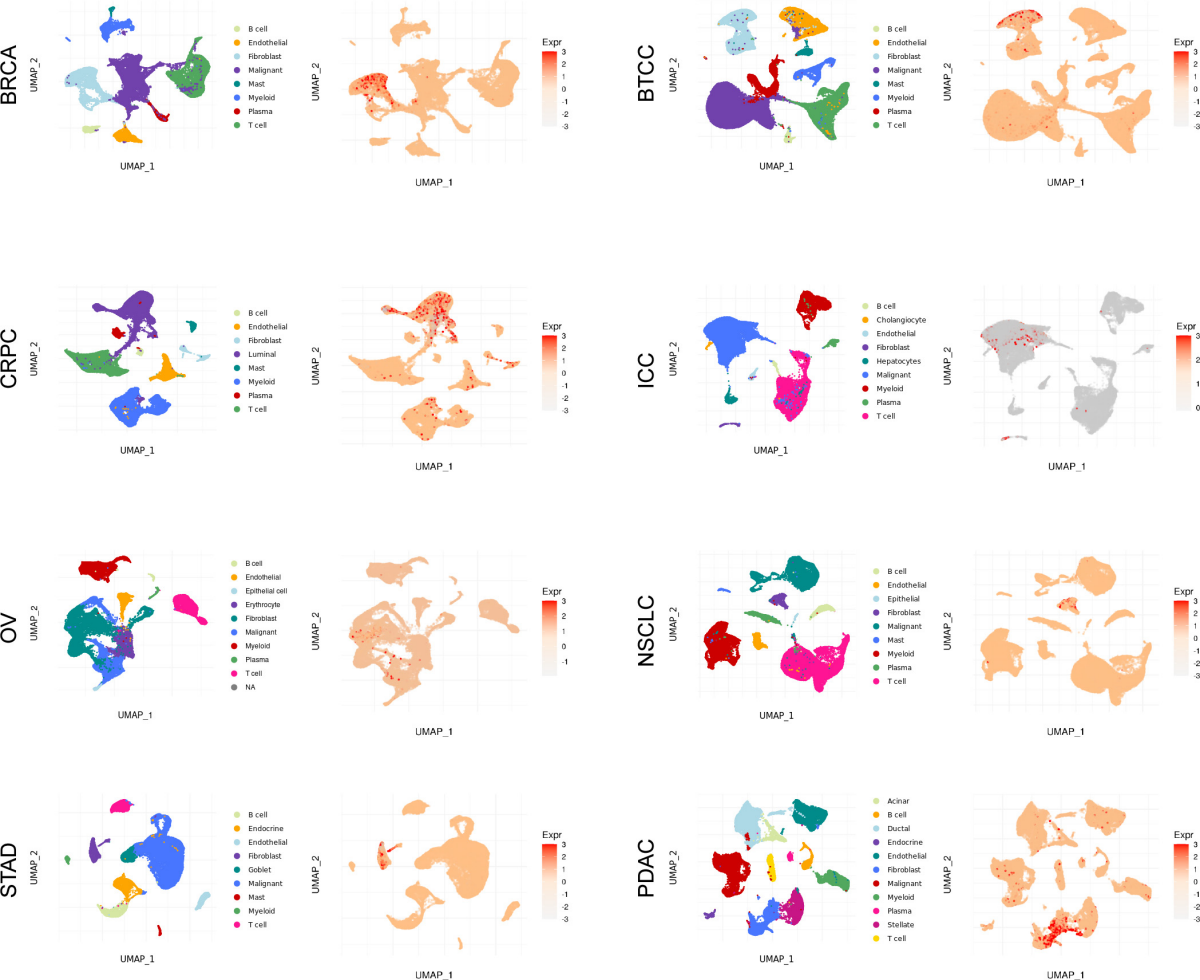
Analysis of single-cell expression distribution patterns of CST2 in pan-cancer.

As depicted in **Figure 2** and **Supplementary Figure S1**, the single-cell analysis results reveal distinct expression patterns of CST2 across various cancer types. Specifically, in BRCA, STAD, BTCC, NSCLC, and PDAC, CST2 exhibits predominantly high expression levels in the Fibroblast cell population. In CRPC, CST2 is primarily highly expressed in the Luminal cell population. On the other hand, in OV and ICC, CST2 shows elevated expression levels mainly in the Malignant cell population.

### Survival analysis

**Figure 3:**
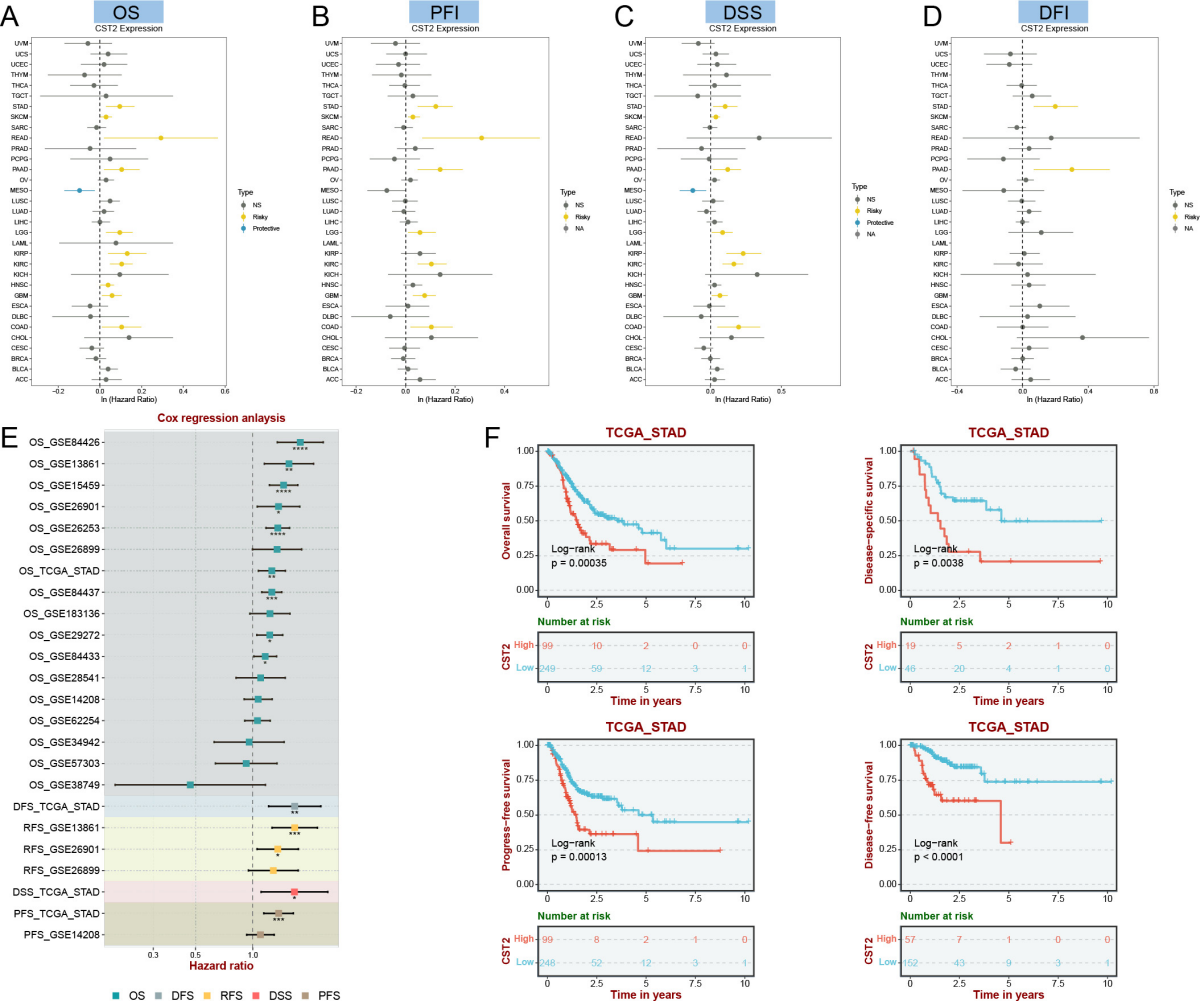
Univariate COX regression analysis was performed to examine the association of CST2 with Overall Survival (OS), Progression Free Interval (PFI), Disease Specific Survival (DSS), and Disease Free Interval (DFI) in pan-cancer. The correlation between CST2 expression and OS **(A)**, PFI **(B)**, DSS **(C)**, and DFI **(D)**. **(E)** Cox regression analysis in STAD from TGCA and GEO database. **(F)** Survival analysis using Kaplan-Meier (KM) curves was conducted to investigate the expression of CST2 and its relationship with OS, DSS, progression-free interval (PFS), and disease-free interval (DFS) in STAD.

**Figure 4:**
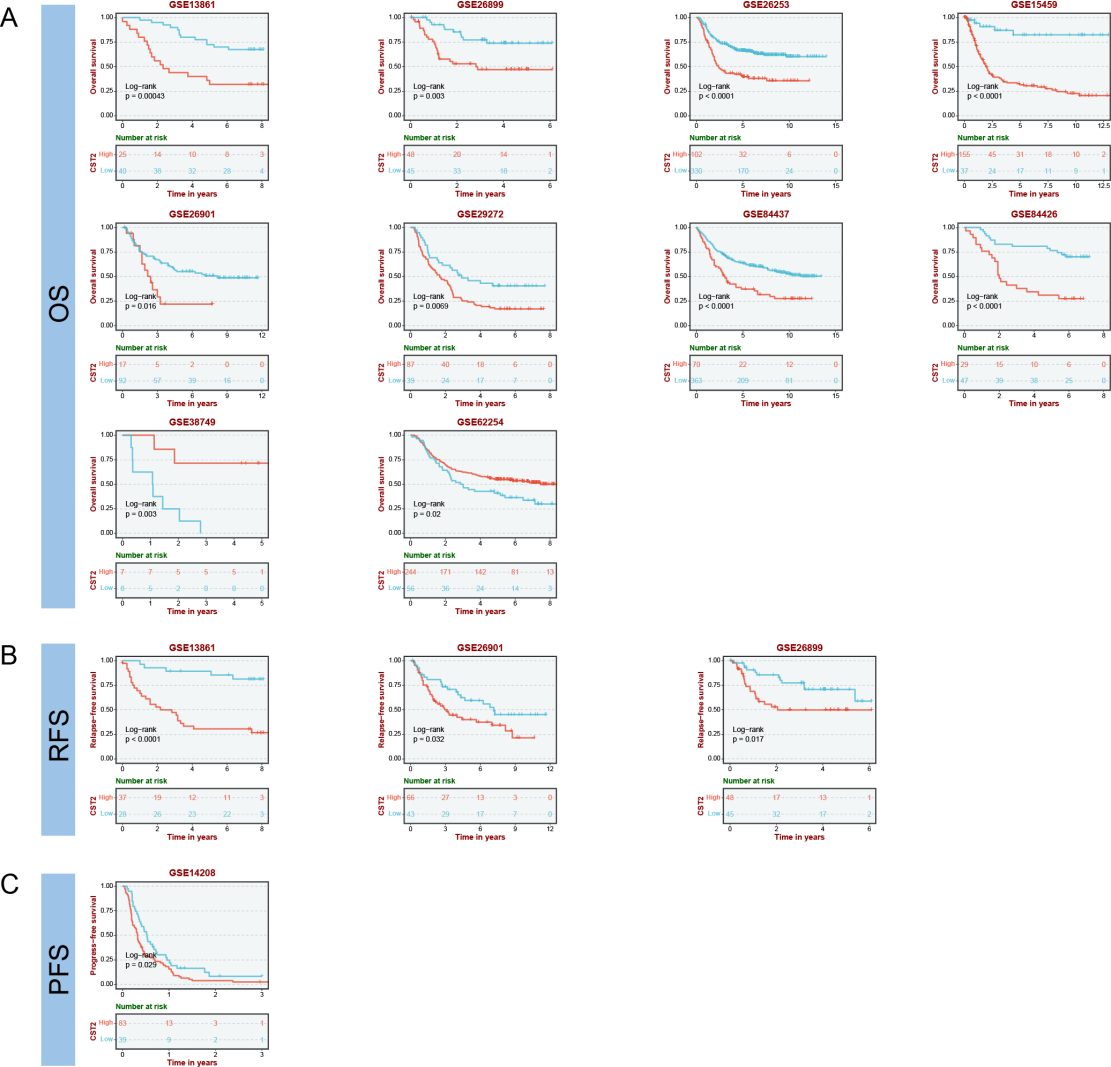
Prognostic analysis of CST2 in the immunotherapy cohort, including OS **(A)**, RFS **(B)** and PFS **(C)**.

COX regression analysis revealed that high expression of CST2 is a risk factor for OS in 10 tumor types, including STAD, SKCM, READ, PAAD, LGG, KIRP, KIRC, HNSC, GBM, and COAD (**Figure 3A**). Further investigations demonstrated a significant correlation between CST2 expression and PFI in several cancer categories, including STAD, SKCM, READ, PAAD, LGG, KIRC, GBM, and COAD (**Figure 3B**). Moreover, elevated expression of CST2 in STAD, SKCM, PAAD, LGG, KIRP, KIRC, GBM, and COAD was associated with improved DSS (**Figure 3C**). Additionally, the univariate Cox regression model established a link between CST2 expression and adverse prognosis in DFI for STAD and PAAD (**Figure 3D**). The survival analysis of CST2 in OS, PFI, DSS, and DFI highlighted its prognostic value across STAD (**Figure 3E**). Furthermore, Kaplan-Meier survival analysis curves were employed to explore the relationship between CST2 expression and OS, DSS, DFS, and PFS specifically in STAD. It was observed that patients with low CST2 expression had better outcomes compared to those with high CST2 expression (**Figure 3F**). The GEO database validated the relationship between CST2 and STAD prognosis in OS (**Figure 4A**), RFS (**Figure 4B**) and PFS (**Figure 4C**).

### Immune cell infiltration

**Figure 5:**
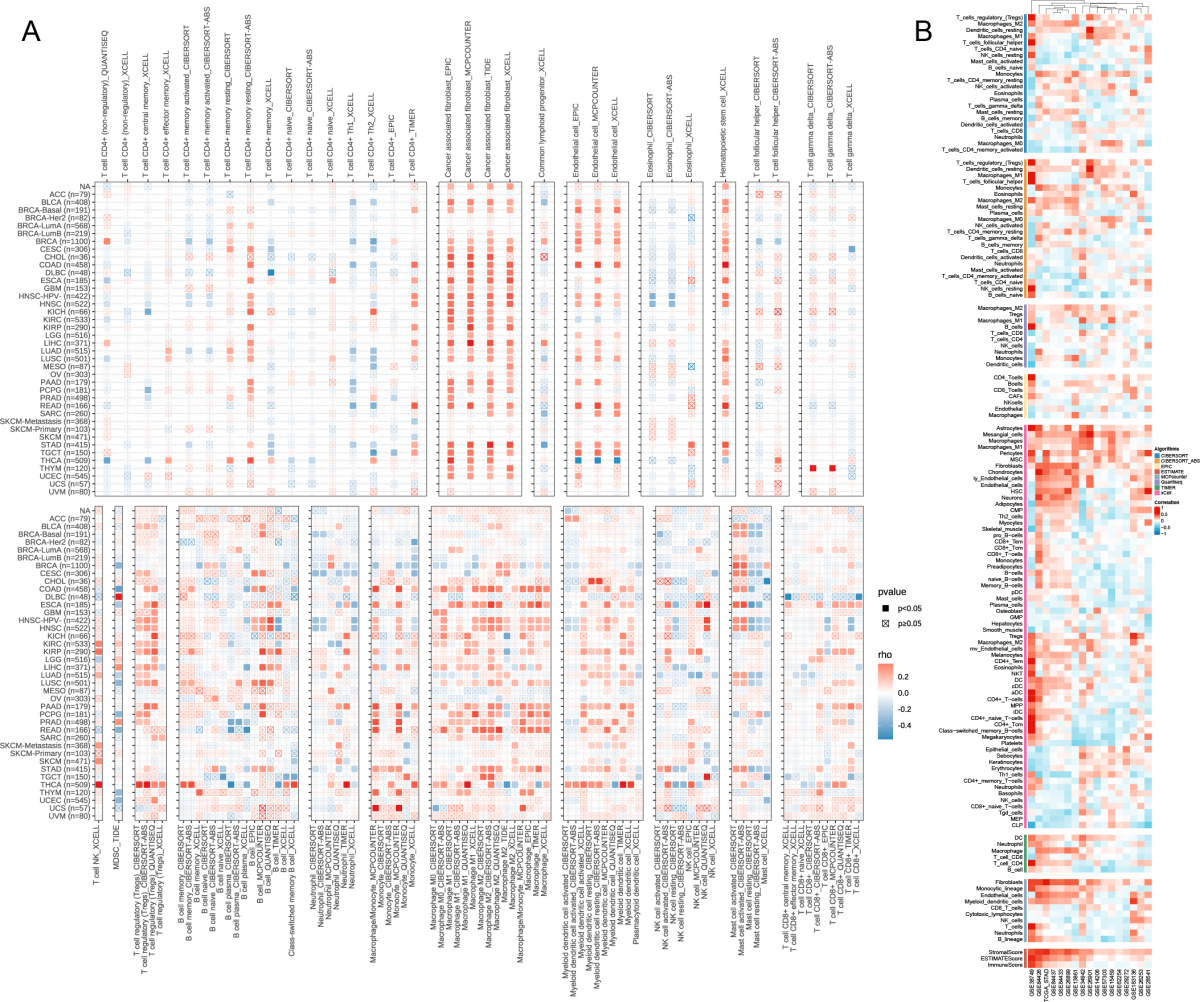
The Relationship of CST2 expression with immune cell infiltration analysis. Immunoinfiltration analysis from Timer2.0 database **(A)** and best database **(B)**.

**Figure 6:**
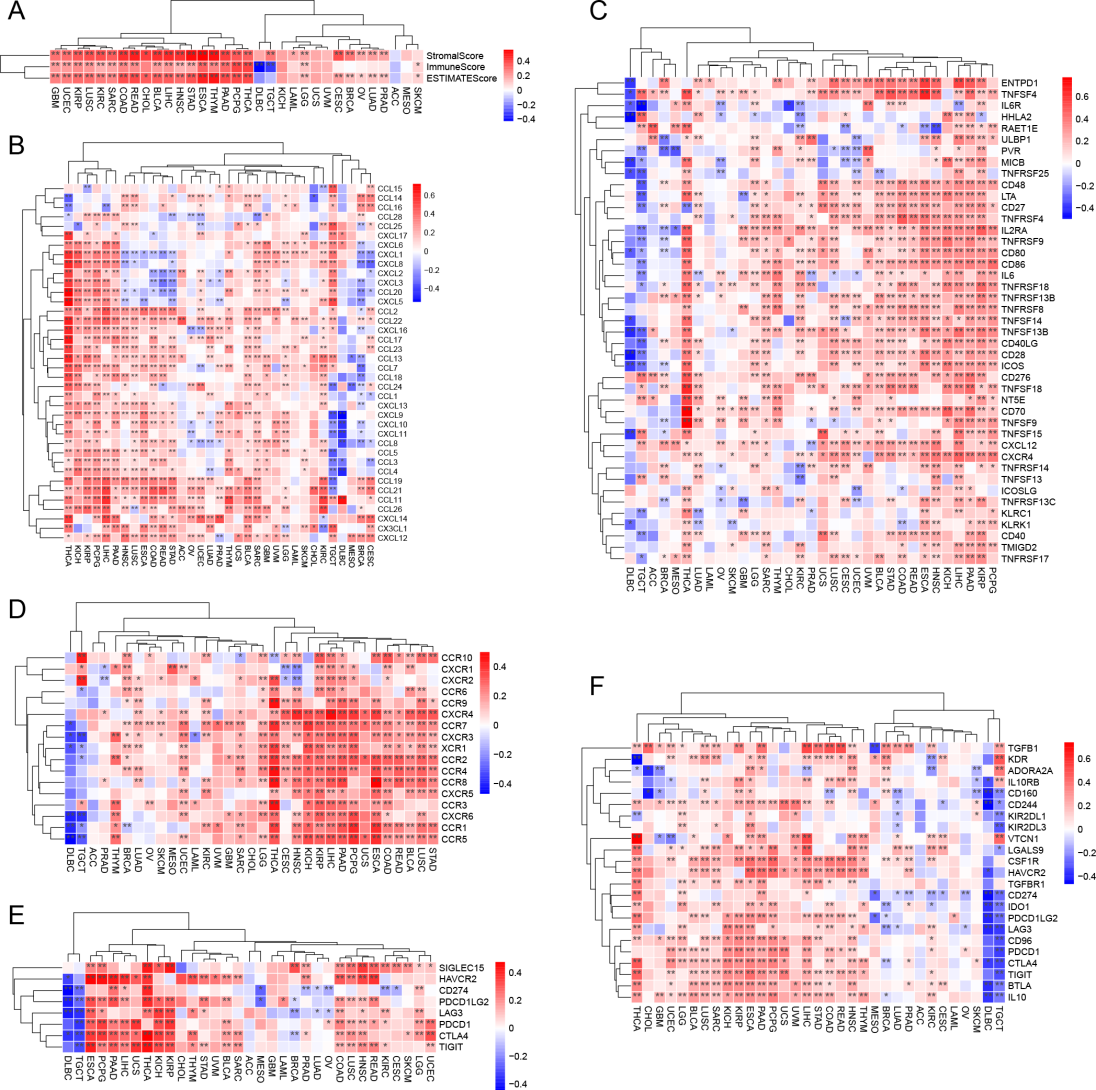
CST2 correlates with immune-related genes. **(A)** Correlation of CST2 with three scores including ESTIMATEScore, ImmuneScore and StromalScore. The Relationship of CST2 expression with **(B)** Chemokine, **(C)** Immunostimulator, **(D)** receptor, **(E)** immune checkpoint genes, and **(F)** Immunoinhibitor (*p < 0.05 and **p < 0.01).

The correlation heatmap reveals that CST2 is closely associated with various immune cells in pan-cancer, including cancer-associated fibroblasts (CAF), endothelial cells, hematopoietic stem cells, regulatory T cells (Tregs), B cells, macrophages, monocytes, myeloid dendritic cells, and CD8+ T cells (**Figures 5A, B**). According to the results obtained from the ESTIMATE algorithm, CST2 exhibits a significant positive correlation with the ESTIMATEScore, ImmuneScore, and StromalScore across different tumor types such as GBM, UCEC, KIRP, LUSC, KIRC, SARC, COAD, READ, BLCA, LIHC, HNSC, STAD, ESCA, THYM, PAAD, PCPG, THCA, and LGG (**Figure 6A**).

### Correlation analysis of CST2 and immune-related genes

The study findings indicate that CST2 exhibits significant positive correlations with several chemokines, such as CCL19, CCL21, CCL11, CCL26, CXCL14, CX3CL1, and CXCL12, in most tumors (**Figure 6B**). Furthermore, CST2 shows significant positive correlations with immunostimulators, including CD48, LTA, CD27, TNFRSF4, IL2RA, TNFRSF9, CD80, and CD86 (**Figure 6C**). Additionally, CST2 demonstrates significant positive correlations with receptors such as CXCR4, CCR7, CXCR3, XCR1, CCR2, CCR4, CCR8, CXCR5, CCR3, CXCR6, CCR1, and CCR5 (**Figure 6D**). Moreover, CST2 exhibits significant positive correlations with immune checkpoint genes, including CD274, CTLA4, HAVCR2, LAG3, PDCD1, PDCD1LG2, SIGLEC15, and TIGIT (**Figure 6E**). Lastly, CST2 shows significant positive correlations with immunoinhibitors like CSF1R, HAVCR2, PDCD1LG2, LAG3, CD96, PDCD1, CTLA4, TIGIT, BTLA, and IL10 (**Figure 6F**).

### The immunotherapeutic potential of CST2

**Figure 7:**
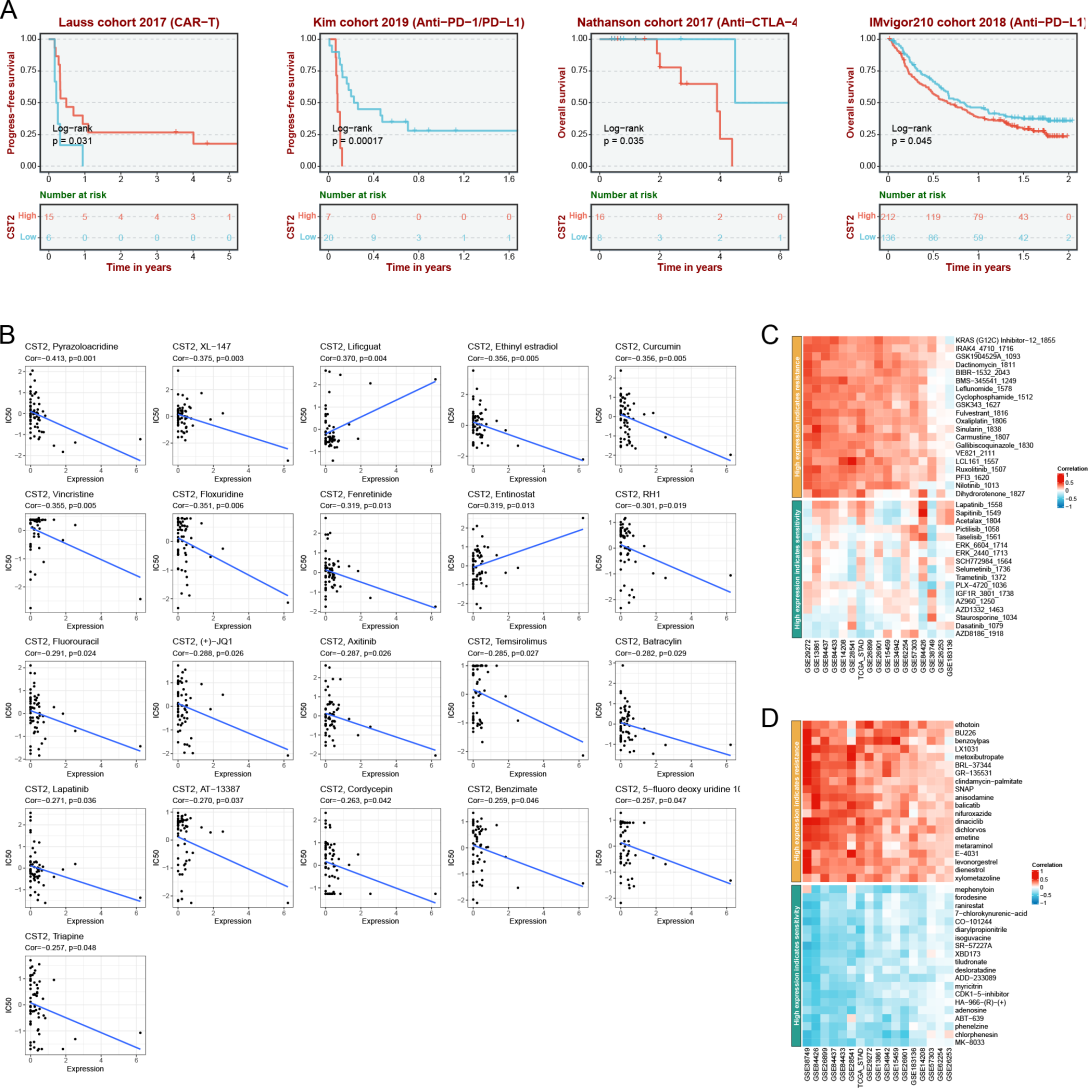
Immunotherapy analysis. **(A)** CST2 expression and prognostic analysis of chimeric antigen receptor-modified T (CAR-T), anti-programmed cell death protein 1/programmed cell death ligand 1 (PD-1/PD-L1), anti-cytotoxic T lymphocyte-associated antigen 4 (CTLA-4), and anti-PD-L1 immunotherapy cohorts. Drug sensitivity analysis of CST2 from cellMiner **(B)**, Genomics of Drug Sensitivity in Cancer (GDSC) database **(C)** and Cancer Therapeutics Response Portal (CTRP) database **(D)**.

Prognostic analysis within the immunotherapy cohorts reveals that high expression of CST2 improves patient prognosis in the Lauss cohort 2017 (CAR-T) (**Figure 7A**). Conversely, low expression of CST2 enhances patient prognosis in the Kim cohort 2019 (Anti-PD-1/PD-L1), Nathanson cohort 2017 (Anti-CTLA-4), and IMvigor210 cohort 2018 (Anti-PD-L1) (**Figure 7A**). Low expression of CST2 enhances prognosis in Anti-PD-1/PD-L1 treatment. Furthermore, drug sensitivity analysis demonstrates a significant association between CST2 and 21 chemotherapy drugs. Examples include Pyrazoloacridine, XL-147, Lificguat, Ethinyl estrdiol, Curcumin, Vincristine, Floxuridine, Fenretinide, Entinostat, RH1, Fluorouracil, (+)-JQ1, Axitinib, Temsirolimus, Batracylin, Lapatinib, AT-13387, Cordycepin, Benzimate, 5-fluoro deoxy uridine, and Triapine (**Figure 7B**). GDSC database (**Figure 7C**) and CTRP database (**Figure 7D**) validate this result.

### Correlation analysis of CST2 with mutations, methylation, TMB, and MSI

**Figure 8:**
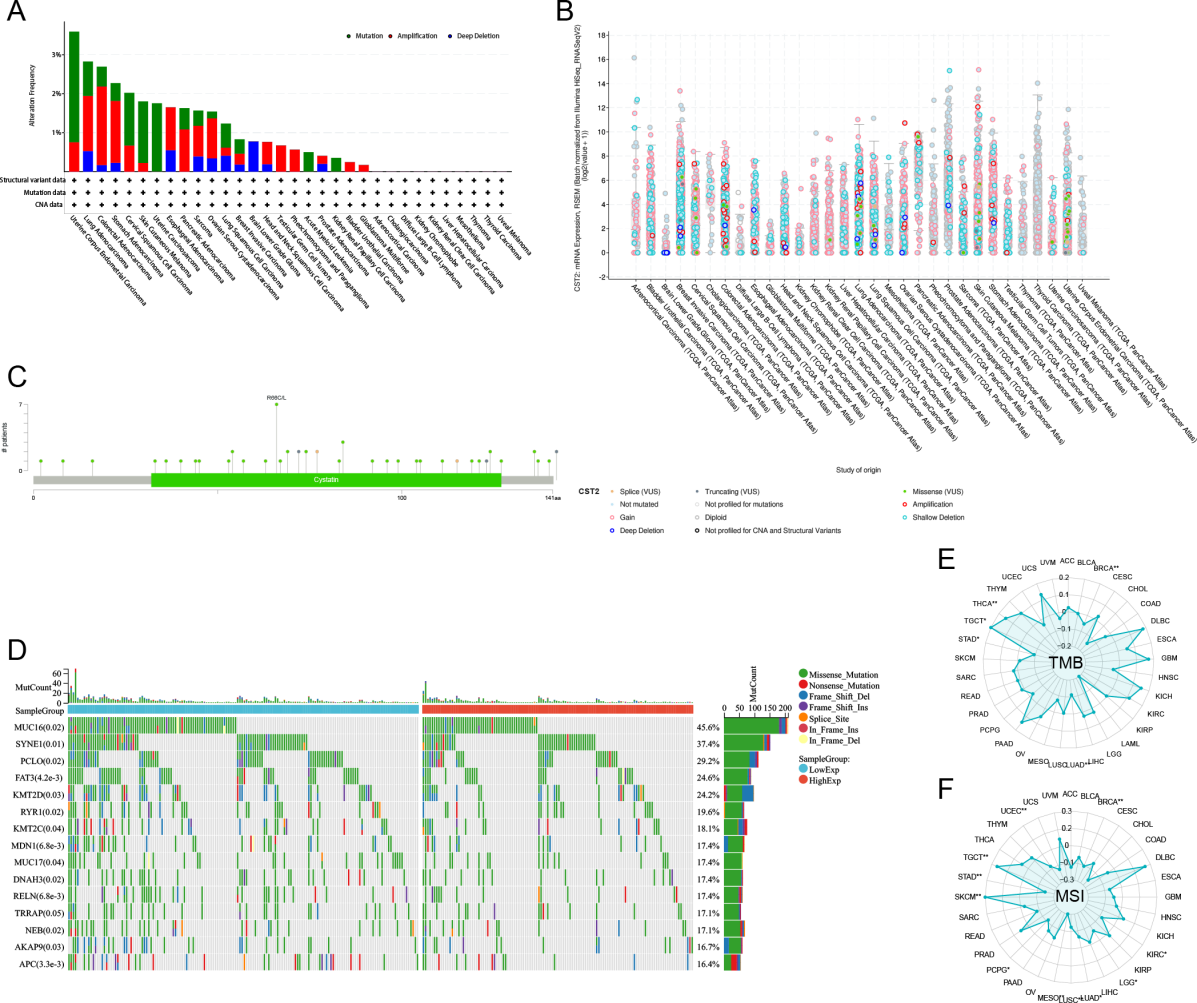
The correlation of CST2 expression with mutation, DNA methylation, tumor mutation burden (TMB), and microsatellite instability (MSI). **(A)** The mutation frequency and corresponding mutation types of CST2 in different cancers. **(B)** The correlation between CST2 expression and DNA methylation. **(C)** Mutation sites of CST2. **(D)** The R package “mafTools” was used to calculate the top 15 genes with the highest mutation frequencies in the low-CST2 (left) and high-CST2 (right) groups of STAD, respectively. Radar plots represent the correlation of CST2 expression with TMB **(E)** and MSI **(F)** in pan-cancer (*p < 0.05 and **p < 0.01).

The predominant mutation type observed is “Mutation,” with the highest mutation frequency of CST2 found in Uterine corpus endometrioid carcinoma (**Figure 8A**). In pan-cancer analysis, a significant correlation is observed between CST2 and methylation (**Figure 8B**). The specific mutation sites of CST2 are illustrated in **Figure 8C**. Furthermore, STAD patients were categorized into two groups based on the median expression level of CST2, and a comparison of gene mutations was conducted between these two groups. The results indicate that patients with low CST2 expression in STAD exhibit a higher frequency of gene mutations compared to those with high CST2 expression (**Figure 8D**). Additionally, **Figure 8E** depicts a significant positive correlation between CST2 and TMB in TGCT and THCA. However, in BRCA, LUAD, and STAD, CST2 exhibits a significant negative correlation with TMB. Regarding MSI, CST2 shows a significant positive correlation with MSI in SKCM and TGCT. Conversely, in BRCA, KIRC, LGG, LUAD, LUSC, MESO, PCPG, STAD, and UCEC, CST2 demonstrates a significant negative correlation with MSI (**Figure 8F**).

### The results of GSEA

**Figure 9:**
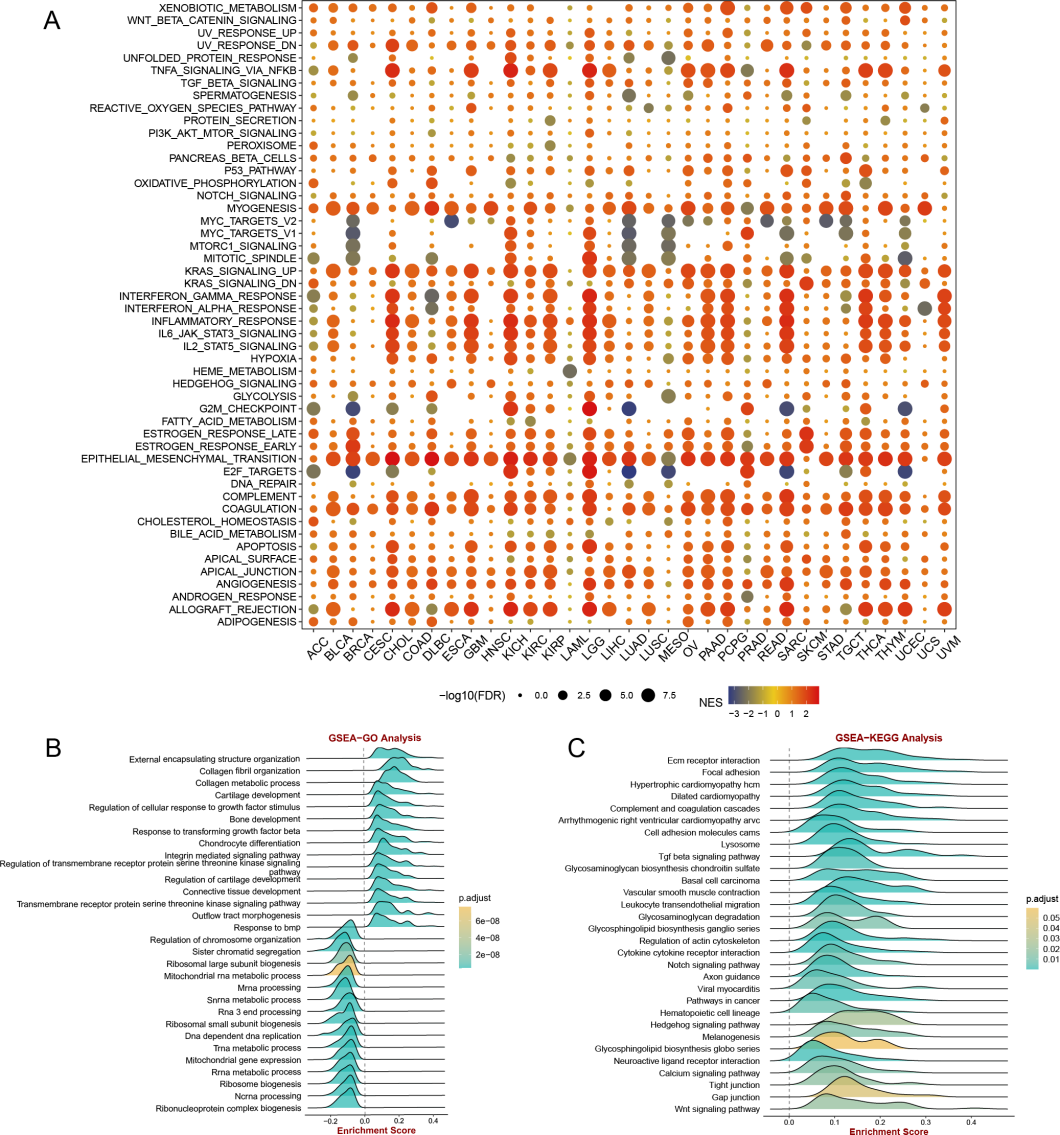
**(A)** gene set enrichment analysis (GSEA) of CST2 in hallmarks gene set. CST2 in STAD for **(B)** Gene Ontology (GO), and **(C)** Kyoto Encyclopedia of Genes and Genomes (KEGG) analysis.

The GSEA results depicted in **Figure 9A** demonstrate that CST2 exhibits a significant positive correlation with various biological processes and signaling pathways in the majority of tumors. Specifically, CST2 shows significant positive correlations with myogenesis, KRAS signaling up, interferon gamma response, interferon alpha response, inflammatory response, IL6 JAK STAT3 signaling, IL2 STAT5 signaling, epithelial-mesenchymal transition, complement, coagulation, apical junction, angiogenesis, allograft rejection, and PI3K AKT mtor signaling (**Figure 9A**). In STAD, GO analysis reveals that CST2 is significantly enriched in External encapsulating structure organization, Collagen fibril organization, Collagen metabolic process (**Figure 9B**). Furthermore, KEGG analysis demonstrates significant enrichment of CST2 in Hedgehog signaling pathway, Glycosphingolipid biosynthesis globo series, Gap junction, and Wnt signaling pathway in STAD (**Figure 9C**).

### Enrichment analysis of CST2 positively and negatively co-expressed genes

**Figure 10:**
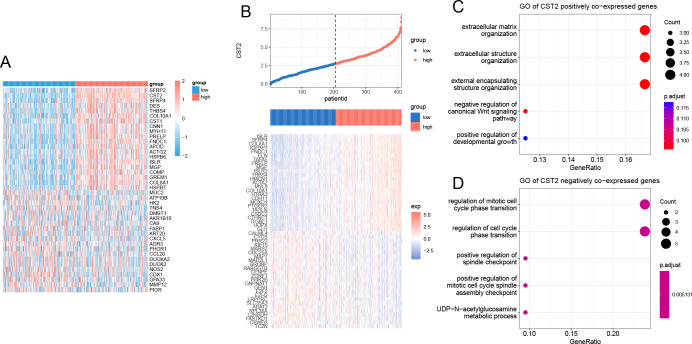
Enrichment analysis of CST2. **(A)** positive and negative co-expression with CST2 in STAD. **(B)** Heatmap. Enrichment analysis of positive **(C)** and negative **(D)** co-expression with CST2.

Applying the Spearman algorithm, we identified the top 20 genes that showed positive and negative co-expression with CST2 in STAD (**Figure 10A**). The heatmap (**Figure 10B**) depicts the expression levels of these genes. Positive co-expressed genes with CST2 were notably enriched in extracellular matrix organization and extracellular structure organization (**Figure 10C**). Conversely, negative co-expressed genes with CST2 were significantly enriched in the regulation of mitotic cell cycle phase transition and regulation of cell cycle phase transition (**Figure 10D**).

### Validating the key role of CST2 in STAD development

**Figure 11:**
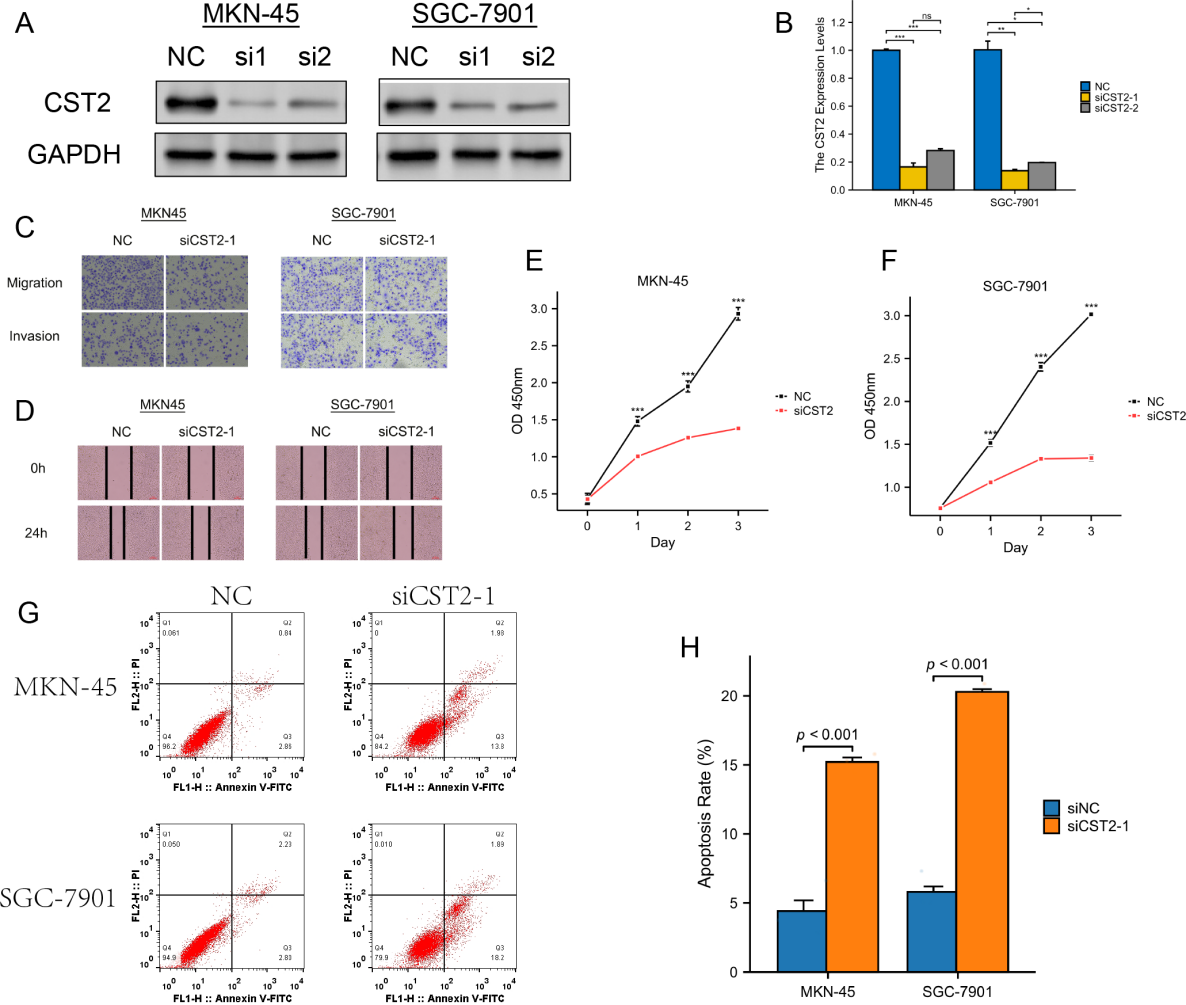
Experimental validation analysis of CST2 in STAD. **(A)** WB showing decreased CST2 expression after CST2 knockout. **(B)** Differential expression analysis. **(C)** Cell migration. **(D-F)** Significant impact on cell cloning afterCST2 knockout. **(G, H)** Apoptosis analysis. Increased levels of apoptosis in both cell lines after CST2 knockdown. *p < 0.05, **p < 0.01, ***p < 0.001.

We selected two STAD cell lines, MKN-45 and SGC-7901, and used gene knockdown techniques to decrease the expression level of CST2. Western blot analysis confirmed a significant reduction in CST2 expression (**Figures 11A, B**). Furthermore, to comprehensively evaluate the effect of CST2 in STAD, we performed transwell migration assays to investigate its impact on cell migration ability. Under conditions of CST2 silencing, cell migration was significantly reduced, which was confirmed by crystal violet staining (**Figure 11C**). To further explore the impact of CST2 on cancer cell proliferation, we conducted colony formation assays. The results demonstrated a significant decrease in the proliferation ability of both cell lines following CST2 knockdown (**Figures 11D–F**), highlighting the crucial role of CST2 in maintaining malignant cell proliferation. Additionally, the levels of apoptosis were significantly increased in both cell lines following CST2 knockdown (**Figures 11G, H**), further supporting the influence of CST2 on cancer cell survival. These findings underscore the central role of CST2 in STAD pathogenesis, influencing disease progression by regulating cancer cell proliferation and migration.

## Discussion

In recent years, mounting evidence has shown the overexpression of CST2 in gastric cancer, colorectal cancer, prostate cancer, hepatocellular carcinoma, and breast cancer. In these cancers, CST2 functions as an oncogene. However, its role in other malignancies remains largely unknown.

Previous studies have demonstrated that overexpression of CST2 contributes to the progression of pancreatic cancer. Analysis of the TCGA and GTEx databases has confirmed abnormally high expression of CST2 in 15 types of tumors. COX regression analysis demonstrates significant correlations between CST2 and OS in STAD, SKCM, READ, PAAD, LGG, KIRP, KIRC, HNSC, GBM, and COAD. Particularly in STAD and PAAD, CST2 exhibits associations with OS, DSS, DFS, and PFS. Further analysis using Kaplan-Meier survival curves demonstrates the prognostic value of CST2 in STAD, where lower expression is associated with better patient outcomes. CST2 shows potential as a reliable biomarker, supported by COX regression analysis and KM survival curves. Additionally, overexpression of CST2 at both mRNA and protein levels is correlated with poor prognosis in late-stage cancer across multiple types, emphasizing its significance in cancer progression. These findings provide direct evidence of CST2’s involvement in cancer initiation and development, solidifying its potential as a target for cancer diagnosis, treatment, and prognosis. However, more research is still needed to confirm the overexpression of CST2 and its oncogenic role in cancer.

Recently, a study has suggested that CST2 may promote the malignant progression of pancreatic cancer through the activation of the PI3K AKT signaling pathway (6). EMT is known to be one of the major factors contributing to tumor cell proliferation, invasion, and metastasis (13). Furthermore, EMT is a crucial factor in the development of drug resistance in cancer treatment (14–16). In gastric cancer, CST2 promotes tumor cell growth, migration, and invasion by modulating EMT and the TGF-β1 signaling pathway (17). Additionally, CST2 may be involved in prostate cancer cell migration through the regulation of the EMT signaling pathway (10). Approximately 1/5 of cancer patients have RAS mutations, which play a significant role in tumorigenesis and progression (18). KRAS is the most common subtype among RAS mutations and is correlated with poor prognosis in cancer patients (19). Interferon gamma levels significantly increase upon stimulation by CST2 (20). In the tumor microenvironment, the IL2 STAT5 signaling pathway can induce CD8+ T cell exhaustion (21).

The tumor microenvironment (TME) is a complex ecosystem consisting of various immune cell types, CAFs, and endothelial cells that surround cancer cells, all embedded within the extracellular matrix (ECM) (22). These cells have been demonstrated to play critical roles in cancer pathogenesis. In our study, we discovered that CST2 is closely associated with multiple immune cells, including CAFs, endothelial cells, hematopoietic stem cells, regulatory T cells (Tregs), B cells, macrophages, monocytes, myeloid dendritic cells, and CD8+ T cells, in pan-cancer. CAFs are an essential cell population in the TME and have been demonstrated to promote tumorigenesis and lead to poorer survival outcomes (23). TAMs are another significant component of the TME, playing roles in coordinating angiogenesis, ECM remodeling, cancer cell proliferation, metastasis, immunosuppression, and resistance to chemotherapy and immune checkpoint blockade therapy (24). CD8+ T cells are potent effector cells that play a crucial role in anti-tumor immune responses, including ICB and adoptive T-cell therapy (25). In cancer, CD4+ T cells exhibit a dual role. Th1 subtype CD4+ T cells contribute to anti-tumor activity by assisting cytotoxic CD8+ T cells and B cells and directly killing cancer cells through interferon and tumor necrosis factor-alpha production. Conversely, Th2 subtype CD4+ T cells secrete anti-inflammatory mediators, promoting tumor growth (26). Tregs, on the other hand, are essential for regulating immune homeostasis and can inhibit effective anti-tumor immunity through various mechanisms (27). Additionally, B cells in cancer exert anti-tumor effects through antibody-dependent cellular cytotoxicity and complement activation (28). These observations highlight the potential role of CST2 in tumor progression by modulating the functions of various immune cells mentioned earlier. Therefore, CST2 represents an attractive therapeutic target in cancer treatment.

Survival analysis demonstrated that high CST2 expression is associated with improved PFS in patients undergoing chimeric antigen receptor-modified T (CAR-T) cell therapy. These findings suggest that patients exhibiting elevated CST2 levels may experience enhanced survival rates following CAR-T treatment. In contrast, low CST2 expression correlates with increased PFS in patients receiving anti-programmed cell death protein 1/programmed cell death ligand 1 (PD-1/PD-L1) therapy, as well as improved OS in those treated with anti-cytotoxic T lymphocyte-associated antigen 4 (CTLA-4) therapy. CAR-T cell therapy represents a groundbreaking approach in adoptive immunotherapy, significantly broadening the horizons for cancer treatment (29). Immune checkpoint proteins, such as PD-1 and PD-L1, are frequently overexpressed in cancer cells and tumor-associated myeloid cells, leading to the suppression of immune surveillance by adaptive immune cells within the TME. Consequently, targeting the PD-1/PD-L1 axis through immune checkpoint blockade (ICB) has emerged as a standard therapeutic strategy for various malignancies (30). Additionally, CTLA-4, another inhibitory immune checkpoint expressed on activated T cells, serves as an effective target for cancer therapy. Agents that inhibit both the PD-1/PD-L1 and CTLA-4 pathways have received approval for the treatment of multiple cancer types. Our research further indicates that among tumor patients receiving CAR-T cell therapy, those with CST2 overexpression are at a lower risk of mortality and demonstrate a better prognosis. Conversely, in patients undergoing anti-PD-1/PD-L1 and anti-CTLA-4 treatments, those with low CST2 expression exhibit more favorable prognoses.

Immunotherapy has become a crucial approach in human anti-tumor treatment, but it has certain limitations (31). Due to the heterogeneity of the TME, immunotherapy is not effective for all cancer patients. The prediction of biomarkers enables more accurate identification of individuals who are likely to benefit from immunotherapy. This precise guidance for treatment helps in determining the appropriate course of action. In tumors, CST2 primarily exhibits mutations at the DNA level. Methylation at the DNA, RNA, and protein levels and their associated downstream signaling pathways participate in various biological processes (32). The use of methylation for cancer diagnosis and treatment is an intriguing research direction. We have observed a significant correlation between CST2 and methylation in pan-cancer. Therefore, CST2 holds promise as a diagnostic marker for detecting mutations and epigenetic alterations across various types of cancer. TMB and MSI are two closely related biomarkers that play important roles in tumor diagnosis, treatment, and prognosis assessment (33, 34). TMB has been used as a predictive biomarker for the efficacy of various immunotherapies, particularly PD-1/PD-L1 inhibitors. Currently, in clinical practice, MSI and TMB are detected to determine if a tumor patient is suitable for immunotherapy and to predict the response and outcome of immunotherapy (35). MSI and TMB, as tumor biomarkers, play significant roles in precision medicine, guiding treatment decisions and improving treatment outcomes. Our study found a significant correlation between CST2 and TMB/MSI in various tumors such as TGCT, THCA, BRCA, LUAD, STAD, SKCM, KIRC, LGG, LUSC, MESO, PCPG, STAD, and UCEC. Thus, CST2 can serve as a predictive biomarker for immunotherapy efficacy in these specific cancers.

In addition, we also analyzed the sensitivity of CST2-related drugs. We identified 21 chemotherapy drugs, including Pyrazoloacridine, XL-147, Lificguat, Ethinyl estradiol, Curcumin, Vincristine, Floxuridine, Fenretinide, Entinostat, RH1, Fluorouracil, (+)-JQ1, Axitinib, Temsirolimus, Batracylin, Lapatinib, AT-13387, Cordycepin, Benzimate, 5-fluoro deoxy uridine, and Triapine, that are associated with CST2 expression. CST2 has the potential to serve as a predictive marker for the efficacy of chemotherapy drugs.

In summary, CST2 is upregulated in various tumor types and is associated with unfavorable prognosis in stomach adenocarcinoma. It is linked to gene mutations, methylation patterns, tumor mutational burden (TMB), microsatellite instability (MSI), immune regulatory genes, immune checkpoint genes, immune cell infiltration, and sensitivity to chemotherapy drugs. While potential molecular mechanisms and related signaling pathways of CST2 have been identified, it is important to note that these findings are primarily based on gastric cancer. Further investigation is necessary to establish whether CST2 can serve as a novel target for cancer diagnosis, treatment, and prognosis across different cancer types, as well as its potential value in predicting the efficacy of anti-tumor immune responses. These insights contribute to a better understanding of the molecular mechanisms underlying CST2’s involvement in tumor initiation and progression, laying the groundwork for future research into targeted therapies and precision medicine.

## Data Availability

The original contributions presented in the study are included in the article/**Supplementary Materials**, further inquiries can be directed to the corresponding authors.

## Supplementary material

Supplementary Figure 1 Analysis of single-cell expression distribution patterns of CST2 in CAC, NNSC, NPC, CCRCC, ESCC and UCEC.

## References

[1] Zhao S Chi H Yang Q Chen S Wu C Lai G. Identification and validation of neurotrophic factor-related gene signatures in glioblastoma and Parkinson’s disease. Front Immunol. (2023) 14:1090040. doi: 10.3389/fimmu.2023.1090040 36825022 PMC9941742

[2] Bagaev A Kotlov N Nomie K Svekolkin V Gafurov A Isaeva O. Conserved pan-cancer microenvironment subtypes predict response to immunotherapy. Cancer Cell. (2021) 39:845–65.e7. doi: 10.1016/j.ccell.2021.04.014 34019806

[3] Brzin J Popovic T Turk V Borchart U Machleidt W. Human cystatin, a new protein inhibitor of cysteine proteinases. Biochem Biophys Res Commun. (1984) 118:103–9. doi: 10.1016/0006-291X(84)91073-8 6365094

[4] Mueller SK Wendler O Nocera A Grundtner P Schlegel P Agaimy A. Escalation in mucus cystatin 2, pappalysin-A, and periostin levels over time predict need for recurrent surgery in chronic rhinosinusitis with nasal polyps. Int Forum Allergy rhinology. (2019) 9:1212–9. doi: 10.1002/alr.22407 31430426

[5] Techatanawat S Surarit R Chairatvit K Roytrakul S Khovidhunkit W Thanakun S. Salivary and serum cystatin SA levels in patients with type 2 diabetes mellitus or diabetic nephropathy. Arch Oral Biol. (2019) 104:67–75. doi: 10.1016/j.archoralbio.2019.05.020 31174096

[6] Ou R Lin C Chen Y. CST2 is activated by RUNX1 and promotes pancreatic cancer progression by activating PI3K/AKT pathway. Arch Biochem biophysics. (2023) 747:109760. doi: 10.1016/j.abb.2023.109760 37722526

[7] Xie Q Liu L Chen X Cheng Y Li J Zhang X. Identification of cysteine protease inhibitor CST2 as a potential biomarker for colorectal cancer. J Cancer. (2021) 12:5144–52. doi: 10.7150/jca.53983 PMC8317524 34335931

[8] Bao Y Wang L Shi L Yun F Liu X Chen Y. Transcriptome profiling revealed multiple genes and ECM-receptor interaction pathways that may be associated with breast cancer. Cell Mol Biol letters. (2019) 24:38. doi: 10.1186/s11658-019-0162-0 PMC6554968 31182966

[9] Zhang WP Wang Y Tan D Xing CG. Cystatin 2 leads to a worse prognosis in patients with gastric cancer. J Biol regulators homeostatic agents. (2020) 34:2059–67. doi: 10.23812/20-293-A 33302616

[10] Song F Zhang Y Pan Z Hu X Yi Y Zheng X. Identification of novel key genes associated with the metastasis of prostate cancer based on bioinformatics prediction and validation. Cancer Cell Int. (2021) 21:559. doi: 10.1186/s12935-021-02258-3 34696780 PMC8547030

[11] Song ZB Yu Y Zhang GP Li SQ. Genomic instability of mutation-derived gene prognostic signatures for hepatocellular carcinoma. Front Cell Dev Biol. (2021) 9:728574. doi: 10.3389/fcell.2021.728574 34676211 PMC8523793

[12] Blanco MA LeRoy G Khan Z Alečković M Zee BM Garcia BA. Global secretome analysis identifies novel mediators of bone metastasis. Cell Res. (2012) 22:1339–55. doi: 10.1038/cr.2012.89 PMC3434351 22688892

[13] Zhang Y Weinberg RA. Epithelial-to-mesenchymal transition in cancer: complexity and opportunities. Front Med. (2018) 12:361–73. doi: 10.1007/s11684-018-0656-6 PMC6186394 30043221

[14] Brabletz T Kalluri R Nieto MA Weinberg RA. EMT in cancer. Nat Rev Cancer. (2018) 18:128–34. doi: 10.1038/nrc.2017.118 29326430

[15] Debaugnies M Rodríguez-Acebes S Blondeau J Parent MA Zocco M Song Y. RHOJ controls EMT-associated resistance to chemotherapy. Nature. (2023) 616:168–75. doi: 10.1038/s41586-023-05838-7 PMC10076223 36949199

[16] Shibue T Weinberg RA. EMT, CSCs, and drug resistance: the mechanistic link and clinical implications. Nat Rev Clin Oncol. (2017) 14:611–29. doi: 10.1038/nrclinonc.2017.44 PMC5720366 28397828

[17] Xie KQ Zhang LM Cao Y Zhu J Feng LY. Adenosine A(1) receptor-mediated transactivation of the EGF receptor produces a neuroprotective effect on cortical neurons *in vitro* . Acta pharmacologica Sin. (2009) 30:889–98. doi: 10.1038/aps.2009.80 PMC4006641 19574994

[18] Prior IA Hood FE Hartley JL. The frequency of ras mutations in cancer. Cancer Res. (2020) 80:2969–74. doi: 10.1158/0008-5472.CAN-19-3682 PMC7367715 32209560

[19] Guo TA Wu YC Tan C Jin YT Sheng WQ Cai SJ. Clinicopathologic features and prognostic value of KRAS, NRAS and BRAF mutations and DNA mismatch repair status: A single-center retrospective study of 1,834 Chinese patients with Stage I-IV colorectal cancer. Int J cancer. (2019) 145:1625–34. doi: 10.1002/ijc.v145.6 PMC6771586 31162857

[20] Kato T Ito T Imatani T Minaguchi K Saitoh E Okuda K. Cystatin SA, a cysteine proteinase inhibitor, induces interferon-gamma expression in CD4-positive T cells. Biol Chem. (2004) 385:419–22. doi: 10.1515/BC.2004.047 15196002

[21] Liu Y Zhou N Zhou L Wang J Zhou Y Zhang T. IL-2 regulates tumor-reactive CD8(+) T cell exhaustion by activating the aryl hydrocarbon receptor. Nat Immunol. (2021) 22:358–69. doi: 10.1038/s41590-020-00850-9 33432230

[22] de Visser KE Joyce JA. The evolving tumor microenvironment: From cancer initiation to metastatic outgrowth. Cancer Cell. (2023) 41:374–403. doi: 10.1016/j.ccell.2023.02.016 36917948

[23] Chen Y McAndrews KM Kalluri R. Clinical and therapeutic relevance of cancer-associated fibroblasts. Nat Rev Clin Oncol. (2021) 18:792–804. doi: 10.1038/s41571-021-00546-5 34489603 PMC8791784

[24] Mantovani A Allavena P Marchesi F Garlanda C. Macrophages as tools and targets in cancer therapy. Nat Rev Drug discovery. (2022) 21:799–820. doi: 10.1038/s41573-022-00520-5 35974096 PMC9380983

[25] Philip M Schietinger A. CD8(+) T cell differentiation and dysfunction in cancer. Nat Rev Immunol. (2022) 22:209–23. doi: 10.1038/s41577-021-00574-3 PMC9792152 34253904

[26] Borst J Ahrends T Bąbała N Melief CJM Kastenmüller W. CD4(+) T cell help in cancer immunology and immunotherapy. Nat Rev Immunol. (2018) 18:635–47. doi: 10.1038/s41577-018-0044-0 30057419

[27] Togashi Y Shitara K Nishikawa H. Regulatory T cells in cancer immunosuppression - implications for anticancer therapy. Nat Rev Clin Oncol. (2019) 16:356–71. doi: 10.1038/s41571-019-0175-7 30705439

[28] Laumont CM Banville AC Gilardi M Hollern DP Nelson BH. Tumour-infiltrating B cells: immunological mechanisms, clinical impact and therapeutic opportunities. Nat Rev Cancer. (2022) 22:414–30. doi: 10.1038/s41568-022-00466-1 PMC9678336 35393541

[29] Sterner RC Sterner RM. CAR-T cell therapy: current limitations and potential strategies. Blood Cancer J. (2021) 11:69. doi: 10.1038/s41408-021-00459-7 33824268 PMC8024391

[30] Zhang H Dai Z Wu W Wang Z Zhang N Zhang L. Regulatory mechanisms of immune checkpoints PD-L1 and CTLA-4 in cancer. J Exp Clin Cancer research: CR. (2021) 40:184. doi: 10.1186/s13046-021-01987-7 34088360 PMC8178863

[31] Zhang Y Zhang Z. The history and advances in cancer immunotherapy: understanding the characteristics of tumor-infiltrating immune cells and their therapeutic implications. Cell Mol Immunol. (2020) 17:807–21. doi: 10.1038/s41423-020-0488-6 PMC7395159 32612154

[32] Dai X Ren T Zhang Y Nan N. Methylation multiplicity and its clinical values in cancer. Expert Rev Mol Med. (2021) 23:e2. doi: 10.1017/erm.2021.4 33787478 PMC8086398

[33] Jardim DL Goodman A de Melo Gagliato D Kurzrock R. The challenges of tumor mutational burden as an immunotherapy biomarker. Cancer Cell. (2021) 39:154–73. doi: 10.1016/j.ccell.2020.10.001 PMC7878292 33125859

[34] Yamamoto H Watanabe Y Maehata T Imai K Itoh F. Microsatellite instability in cancer: a novel landscape for diagnostic and therapeutic approach. Arch toxicology. (2020) 94:3349–57. doi: 10.1007/s00204-020-02833-z 32632538

[35] Palmeri M Mehnert J Silk AW Jabbour SK Ganesan S Popli P. Real-world application of tumor mutational burden-high (TMB-high) and microsatellite instability (MSI) confirms their utility as immunotherapy biomarkers. ESMO Open. (2022) 7:100336. doi: 10.1016/j.esmoop.2021.100336 34953399 PMC8717431

