# Supplementary figures and images for: Pan-cancer and experimental analyses reveal the immunotherapeutic significance of CST2 and its association with stomach adenocarcinoma proliferation and metastasis
Source: Front Immunol. 2025 Jan 24;15:1466806. doi: 10.3389/fimmu.2024.1466806 (PMC11802563; doi:10.3389/fimmu.2024.1466806)

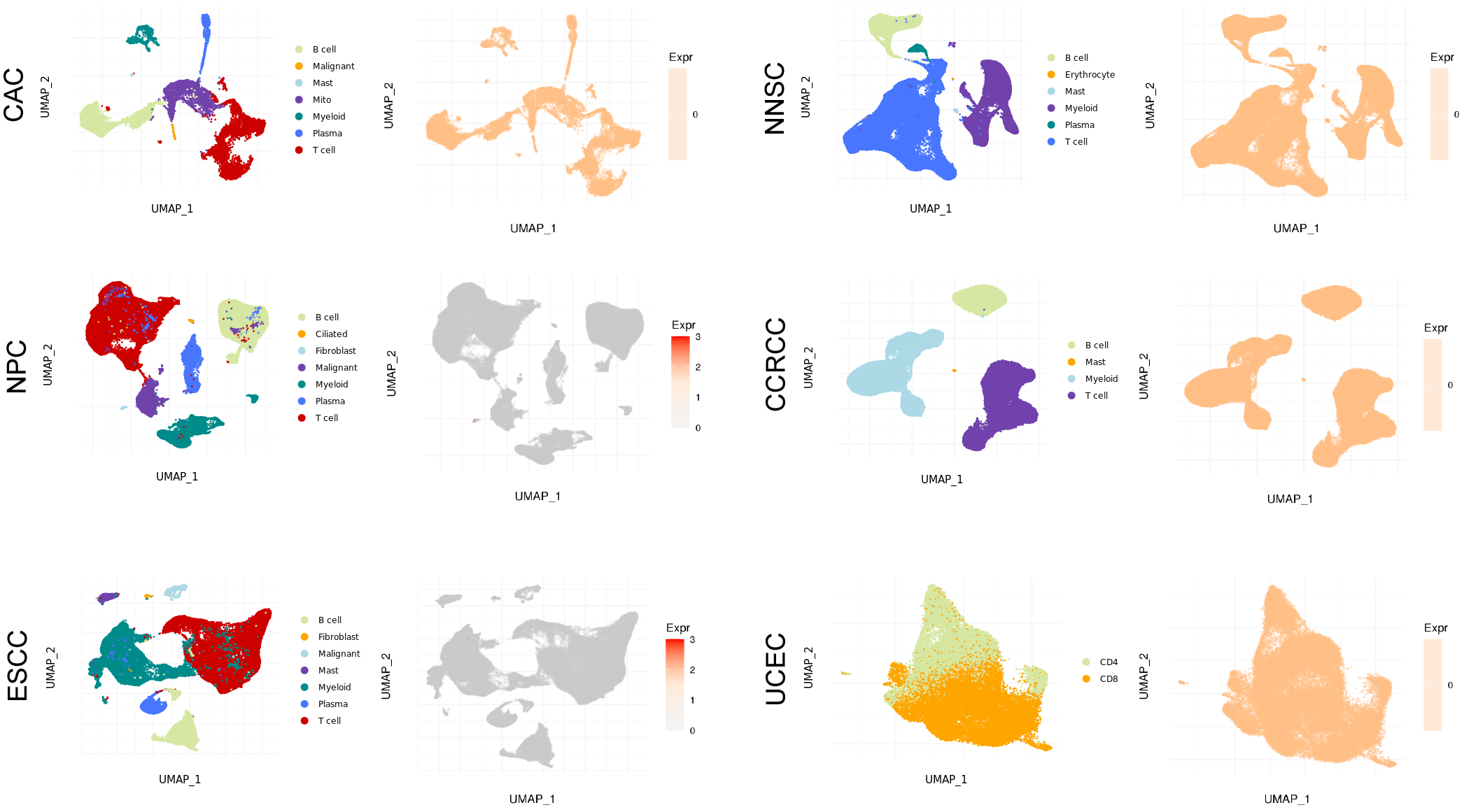

Supplement: Supplementary Figure 1 — Analysis of single-cell expression distribution patterns of CST2 in CAC, NNSC, NPC, CCRCC, ESCC and UCEC. [file Image1.tif]
